# Heart Sound Classification for Early Detection of Cardiovascular Diseases Using XGBoost and Engineered Acoustic Features

**DOI:** 10.3390/s26020630

**Published:** 2026-01-17

**Authors:** P. P. Satya Karthikeya, P. Rohith, B. Karthikeya, M. Karthik Reddy, Akhil V M, Andrea Tigrini, Agnese Sbrollini, Laura Burattini

**Affiliations:** 1 Amrita School of Artificial Intelligence, Coimbatore, Amrita Vishwa Vidyapeetham, Amrita University, Coimbatore 641112, India; cb.ai.u4aid23128@cb.students.amrita.edu (P.P.S.K.); cb.ai.u4aid23131@cb.students.amrita.edu (P.R.); cb.ai.u4aid23109@cb.students.amrita.edu (B.K.); cb.ai.u4aid23123@cb.students.amrita.edu (M.K.R.); vm_akhil@cb.amrita.edu (A.V.M.); 2 Department of Information Engineering, Università Politecnica delle Marche, 60131 Ancona, Italy; a.tigrini@staff.univpm.it (A.T.); a.sbrollini@staff.univpm.it (A.S.)

**Keywords:** heart sound classification, cardiovascular disease detection, machine learning, deep learning

## Abstract

Heart sound-based detection of cardiovascular diseases is a critical task in clinical diagnostics, where early and accurate identification can significantly improve patient outcomes. In this study, we investigate the effectiveness of combining traditional acoustic features and transformer-based Wav2Vec embeddings with advanced machine learning models for multi-class classification of five heart sound categories. Ten engineered acoustic features, i.e., Log Mel, MFCC, delta, delta-delta, chroma, discrete wavelet transform, zero-crossing rate, energy, spectral centroid, and temporal flatness, were extracted as regular features. Four model configurations were evaluated: a hybrid CNN + LSTM and XGBoost trained with either regular features or Wav2Vec embeddings. Models were assessed using a held-out test set with hyperparameter tuning and cross-validation. Results demonstrate that models trained on regular features consistently outperform Wav2Vec-based models, with XGBoost achieving the highest accuracy of 99%, surpassing the hybrid model at 98%. These findings highlight the importance of domain-specific feature engineering and the effectiveness of ensemble learning with XGBoost for robust and accurate heart sound classification, offering a promising approach for early detection and intervention in cardiovascular diseases.

## 1. Introduction

Cardiovascular diseases (CVDs) include a broad spectrum of pathological conditions (e.g., valve disorders or genetic heart diseases), many of which remain undetected until advanced stages [1,2]. Several experts have reported that the impact of CVD will continue to rise in the coming years [3], and its alarming frequency implies the critical need for effective early detection and continuous monitoring of heart functionality to prevent serious conditions [3,4]. In this scenario, advancement in the definition of novel advanced technical applications in cardiovascular diagnosis (e.g., intravascular ultrasound imaging) is mandatory. As screening solutions, technologies based on auscultation may play a key role in monitoring heart conditions, considering the wide spectrum of clinical knowledge of heart sounds using the stethoscope, and its simple and noninvasive approach.

The heart sounds, specifically S1 and S2, stand as indicators of heart health, specifically the functionality of heart valves and the entire cardiac cycle [5]. The S1 is produced by the closing of the mitral and tricuspid valves at the beginning of ventricular systole, while the S2 occurs when the aortic and pulmonary valves close at the end of ventricular systole [5]. Anomalies associated with S1 changes can be signs of mitral stenosis or regurgitation, while anomalies associated with S2 changes may indicate pulmonary stenosis or embolism. In addition to these primary heart sounds, S3 and S4 may also be present, indicating, for example, the presence of heart failure or hypertrophic ventricle, respectively. All four types of sounds may contribute to clinical evaluation and are particularly useful in drawing attention when specific alterations in typical sound patterns are heard by the physician [6].

Despite being widely used, auscultation remains a highly user-dependent technique [7]. Heart and lung sounds can be difficult to detect, and variations in stethoscope placement or applied pressure can significantly affect what is perceived. The quality of the stethoscope and the presence of environmental noise further influence diagnostic accuracy, while patient-related factors such as obesity or hyperinflated lungs may attenuate the sounds. Moreover, the early detection of cardiovascular diseases using auscultation alone is particularly challenging, as faint pathological sounds may be overlooked, and auditory perception can vary depending on clinicians’ fatigue, workload, and individual experience levels [3]. These limitations underscore the need for an unbiased and automated approach to heart sound analysis, which can minimize subjective errors and enhance the reliability and efficiency of the detection process [3,8]. These drawbacks underscore the immediate need for more reliable and objective diagnosis tools, which drives the development of automated systems for heart-sound classification using advanced signal processing and artificial intelligence (AI) techniques. These decision support systems could offer tools for structuring information and features extracted from heart sounds, and for developing models aimed at the early recognition of heart anomalies, alerting clinicians and supporting their work during daily screening. Thus, these systems can help reduce the errors due to subjectivity, that may arise when a large number of patients are evaluated throughout the day [4,8].

Among the many techniques for heart sound classification, there are the traditional methods and the AI-powered ones. Traditional methods often rely on technical expertise, which leads to variable interpretations of heartbeats, while extensive research has been conducted in the field of AI for medical diagnosis, particularly related to the heart. For AI models to accurately diagnose heart conditions, feature extraction is essential. Feature extraction plays a crucial role in classifying heart sounds, as raw recordings alone are insufficient for predicting heart abnormalities [5,8]. For effective classification, relevant features must be extracted from the heart sound data. Several feature extraction techniques have been developed based on time-domain, frequency-domain, and time–frequency domain representations of signals, all capable of capturing essential characteristics. Among the most commonly used techniques are the Mel Frequency Cepstral Coefficient (MFCC) and Log-Mel [X]. MFCC captures the frequency content of the sound while reducing unnecessary noise. Additionally, two derivative features can be extracted, i.e., delta and delta-delta, which represent the first and second derivatives of MFCC, corresponding to its velocity and acceleration [9,10]. Log-Mel, on the other hand, is derived from the Mel spectrogram, an energy representation of sound in the frequency domain. Spectrograms are expressed along the time and frequency axes [11]. The extracted features serve as inputs for AI models to classify different heart conditions. These features provide information about temporal variations, spectral properties, and frequency patterns, enabling models to distinguish between normal and abnormal heart sounds [12]. Machine learning (ML) algorithms such as decision trees, random forests, and support vector machines analyze large volumes of data to identify patterns and relationships that are often difficult for humans to discern manually [13].

Deep learning (DL) techniques, such as Convolutional Neural Networks (CNNs) and Long Short-Term Memory (LSTM) networks, have significantly advanced the classification of heart diseases. These approaches analyze heart sound recordings by leveraging the strengths of both architectures: CNNs are effective at extracting spatial features from time–frequency representations of heart sounds, such as spectrograms, while LSTMs excel at capturing temporal dependencies present in sequential data [12,14,15]. Unlike traditional ML algorithms, which learn patterns from data in the feature space to make predictions without explicit programming, DL models rely on neural networks with multiple layers capable of automatically learning complex feature representations [16].

The spatial patterns of heart sounds often relate to hemodynamic disturbances caused by stenosis or regurgitation. When these characteristics are extracted, the LSTM module captures their progression over time during the cardiac cycle, encoding timing, rhythm, and duration of sounds such as S1 and S2, as well as irregular third and fourth (S3 and S4) sounds. Encoding this temporal progression is crucial, as many cardiac disorders manifest through changes in timing and regularity of valve closures or turbulent blood flow. By integrating both spatial and temporal information, the CNN–LSTM model emulates the diagnostic reasoning of an experienced clinician, enabling accurate discrimination between healthy and diseased cases. This hybrid model not only improves classification accuracy but also demonstrates robustness in real clinical scenarios, where heart sounds are often weak and highly variable [16].

Recent advances, such as the SS-LSTM model, further highlight the importance of capturing multi-scale information [17]. By employing hierarchical LSTM networks, these approaches enhance accuracy in complex contexts, such as trajectory prediction in crowded environments [17]. Similarly, integrating CNNs with LSTMs produces models capable of extracting spatial features while simultaneously capturing sequential temporal relationships [18].

In addition to DL approaches, traditional ML models such as XGBoost remain valid and effective options for classification tasks [19]. Although XGBoost is considered a shallow model compared to deep neural networks, it can achieve competitive performance when trained with carefully engineered features. In particular, XGBoost is well-suited for handling imbalanced datasets through regularization techniques, and it has delivered state-of-the-art results across diverse applications, including those involving complex, low-frequency signal data [20]. The algorithm introduces several innovations, such as a sparsity-aware method for efficiently handling sparse data and a weighted quantile sketch technique that enables approximate learning in large datasets. Moreover, XGBoost can process large volumes of samples while requiring fewer computational resources than deep models, making it a practical choice in real-world ML scenarios where both speed and accuracy are critical [19,20].

In this context, the present study investigates the effectiveness of combining conventional acoustic features with advanced transformer-based representations for heart sound classification. By emphasizing the role of efficient feature extraction, the proposed approach evaluates how different feature combinations impact the performance of XGBoost classifier in detecting cardiovascular conditions such as aortic stenosis, mitral stenosis, mitral regurgitation, and mitral valve prolapse. This design choice enables accurate classification with reduced computational complexity, making the proposed machine learning framework particularly suitable for deployment in embedded and resource-constrained healthcare systems.

## 2. Materials and Methods

### 2.1. Dataset Presentation

The dataset used in this work was obtained from a publicly available repository [5]. The heart sound recordings were collected using an electronic stethoscope and categorized into five classes: Aortic Stenosis (AS), Mitral Regurgitation (MR), Mitral Valve Prolapse (MVP), Mitral Stenosis (MS), and Normal (N) [5]. The dataset contains a total of 1000 recordings, with 200 recordings per class. Each recording has an average duration of 2.44 s, covering at least one complete cardiac cycle.

**Figure 1:**
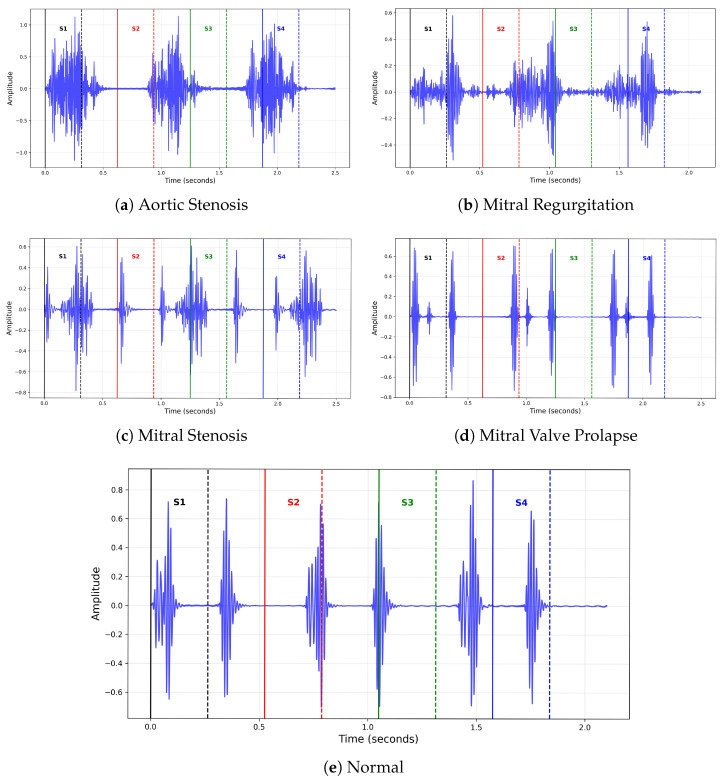
Heart Sound Patterns for Different Conditions.

The recordings capture characteristic acoustic patterns of each class: AS recordings exhibit high-frequency systolic murmurs caused by narrowed aortic valves; MR recordings contain systolic murmurs produced by retrograde blood flow through the mitral valve; MVP recordings typically feature mid-systolic clicks associated with murmurs; MS recordings include low-frequency diastolic murmurs; and the Normal class consists of pure heart sounds without pathological murmurs [5]. An example of typical signal epochs from the dataset is shown in Figure 1.

All audio recordings were subjected to a standardized preprocessing pipeline to ensure uniformity in sampling rate, amplitude, and signal duration before feature extraction and model training. Specifically, all .wav files were resampled to a common sampling rate of 8 kHz, followed by amplitude normalization using min–max scaling. To suppress baseline drift and high-frequency noise, a 4th-order bidirectional Butterworth band-pass filter with cutoff frequencies of 20–4000 Hz was applied. Subsequently, the signals were segmented into fixed-length 3 s windows covering complete cardiac cycles, with zero-padding employed for recordings shorter than the target duration. This structured preprocessing strategy ensures consistent input representations and provides a robust foundation for the reliable development and evaluation of machine learning models for automatic heart disease classification [5].

### 2.2. Pattern Recognition Experimental Design and Validation Strategy

To rigorously assess model generalizability and prevent data leakage, the dataset was stratified into three independent subsets: 70% for training, 15% for validation, and 15% for testing. Crucially, this partition was performed at the subject level. Since each recording in the dataset corresponds to a unique patient, this split ensures that no subject data appears in more than one subset, thereby adhering to the strict subject-independence requirement for medical diagnostic models [21]. The validation set was utilized exclusively for hyperparameter optimization (e.g., learning rate, tree depth) and early stopping, while the Test set was held out as unseen data for the final performance verification.

To evaluate the statistical stability of the models, a robust cross-validation scheme was used within the 70% Training partition. For the hybrid CNN-LSTM architecture, we utilized a 10-fold cross-validation (N=10) to evaluate convergence stability across varying data folds. Conversely, for the XGBoost model, we applied a Monte Carlo cross-validation with 20 independent repetitions (N=20) to assess the robustness of the gradient-boosted ensemble.

The statistical comparison of these performance distributions was conducted using the Wilcoxon Rank-Sum test. This non-parametric hypothesis test was selected specifically for its ability to compare independent samples without assuming normal distribution, ensuring that the reported stability differences between the deep learning and ensemble approaches are statistically significant.

### 2.3. Feature Definition and Extraction

#### 2.3.1. Log-Mel

Log-Mel is commonly used to approximate how humans perceive sound. It emphasizes lower frequencies more than higher ones, reflecting the fact that human hearing is more sensitive to lower frequencies [22]. The mathematical representation is as follows:

(1) $$X_{P}\;=\;\log\left(M|S|\right)$$

where $X_{P}$ is the Log-Mel spectrogram, *M* is the Mel filter bank, and *S* is the spectrogram of the audio signal.

#### 2.3.2. Mel Frequency Cepstral Coefficients (MFCC)

MFCCs highlight the low-frequency components of a signal while also capturing higher frequencies, which aids in distinguishing between different sounds. MFCCs help focus on relevant components of the signal and reduce redundancy by treating correlated bands as noise [9,23].

(2) $$C_{i}\;=\;\sum_{n=1}^{N_{f}}S_{n}\cos\left(i\left(n-0.5\right)\left(\frac{\pi }{N_{f}}\right)\right)$$

Here, $C_{i}$ is the *i*-th cepstral coefficient, $S_{n}$ is the *n*-th filter coefficient of the log-energy output of the signal, and $N_{f}$ is the number of filters.

#### 2.3.3. Delta and Delta-Delta

Delta and Delta-Delta are the first and second derivatives of MFCCs, respectively. Delta captures the rate of change of MFCCs over time, while Delta-Delta represents the acceleration of spectral features. Together, they enhance model robustness by emphasizing dynamic differences between normal and abnormal sounds [24].

#### 2.3.4. Chroma

Chroma features capture the energy distribution across 12 pitch classes associated with musical notes. By identifying periodicity and patterns in the signal, Chroma features can highlight different states of heart valve activity.

(3) $$C\left(m,c\right)\;=\;\sum Y_{\mathrm{LF}}\left(m,p\right)$$

Here, C(m,c) is the Chroma feature, and $Y_{\mathrm{LF}}$ is the absolute energy value corresponding to frame *m* and pitch *p*.

#### 2.3.5. Zero-Crossing Rate (ZCR)

ZCR measures how frequently a signal changes sign from positive to negative or vice versa. It is useful for identifying rapid variations in the signal.

(4) $$\mathrm{zcr}\;=\;\frac{1}{T-1}\sum_{t=1}^{T-1}1_{R<0}\left(s_{t}\cdot s_{t-1}\right)$$

Here, zcr is the zero-crossing rate, $s_{t}$ is the signal at time *t*, and $1_{R<0}$ is an indicator function.

#### 2.3.6. Discrete Wavelet Transform (DWT)

DWT provides joint analysis in both the time and frequency domains by using wavelets—small oscillatory functions with finite energy. Unlike traditional Fourier basis functions, wavelets can capture both high- and low-frequency components simultaneously [25]. DWT decomposes the signal into approximation and detail components, making it effective for robust feature extraction [26].

(5) $$y\left[n\right]\;=\;\left(x*g\right)\left[n\right]\;=\;\sum_{k=-\infty }^{\infty }x\left[k\right]g\left[n-k\right]$$

Here, y[n] represents the transformed signal (DWT coefficients), x[k] is the original signal at index *k*, g[n−k] is the wavelet function applied at shift n−k, and ∗ denotes convolution.

#### 2.3.7. Spectral Contrast

Spectral Contrast measures the amplitude difference between spectral peaks and valleys. It provides information about the tonal quality of sounds and is particularly useful for distinguishing pathological murmurs in cardiovascular conditions.

(6) $${\mathrm{sc}}_{k}\;=\;\mathrm{mean}\left(P_{k}\right)-\mathrm{mean}\left(v_{k}\right)$$

Here, ${\mathrm{sc}}_{k}$ is the spectral contrast, $P_{k}$ is the amplitude of peaks, and $v_{k}$ is the amplitude of valleys.

#### 2.3.8. Spectral Centroid

The Spectral Centroid indicates the “center of mass” of the spectrum, showing where most of the energy is concentrated. Shifts in the centroid can reflect abnormalities in heart sound frequency content.

(7) $$C\;=\;\frac{\sum_{n=0}^{N-1}f\left(n\right)x\left(n\right)}{\sum_{n=0}^{N-1}x\left(n\right)}$$

Here, x(n) is the weighted frequency value, and f(n) is the center frequency of the bin.

#### 2.3.9. Energy

Energy represents the total power of a signal over time, distinguishing loud from quiet heart sounds. This feature helps identify abnormal variations in intensity.

(8) $$E\;=\;\sum_{t=1}^{T}{\left|x\left(t\right)\right|}^{2}$$

Here, *E* is the energy, x(t) is the signal at time *t*, and *T* is the total time.

#### 2.3.10. Temporal Flatness

Temporal Flatness measures how evenly energy is distributed across time. It indicates whether a sound is more noise-like or tonal, helping detect irregularities in heart sounds.

(9) $$\mathrm{TF}\;=\;\frac{{\left(\mathrm{mean}(|x|)\right)}^{2}}{\mathrm{mean}(|x{|}^{2})}$$

Here, TF is the temporal flatness, and *x* represents signal values.

### 2.4. Transformer-Based Feature Extraction (Wav2Vec)

The Wav2Vec transformer leverages DL for speech representation learning, extracting meaningful features directly from raw audio waveforms [22]. Wav2Vec employs convolutional layers to capture low-level features such as amplitude variations, followed by a transformer-based context network that models higher-level representations and long-range dependencies [27].

Wav2Vec 2.0, a self-supervised transformer-based model, was employed in this study to extract contextualized features from heart sound recordings. Let the input signal x(t) represent the raw waveform sampled at 16 kHz. The waveform is first encoded using 1D convolutional layers $f_{\mathrm{conv}}$, which transform it into a sequence of latent representations $z\in R^{T\times d}$, where *T* is the number of frames and *d* is the dimensionality (e.g., 768 for Wav2Vec2-Base):

(10) $$z\;=\;f_{\mathrm{conv}}\left(x\left(t\right)\right)$$

These latent features are then passed through a multi-layer Transformer encoder $f_{\mathrm{transformer}}$, which applies self-attention and positional encoding to capture long-range temporal dependencies:

(11) $$h\;=\;f_{\mathrm{transformer}}\left(z\right),\;h\in R^{T\times d}$$

Here, *h* denotes the contextualized representations at each time step. To convert *h* into a fixed-length feature vector suitable for classification, the sequence is flattened:

(12) $$\hat{h}\;=\;\mathrm{flatten}\left(h\right)\in R^{T\cdot d}$$

Since $\hat{h}$ varies with input duration, it is padded or truncated to a fixed target length *L* (here, L=2400):

(13) $${\hat{h}}_{\mathrm{final}}\;=\;\mathrm{pad\_or\_truncate}\left(\hat{h},L\right)$$

The final vector ${\hat{h}}_{\mathrm{final}}$ is concatenated with traditional acoustic features such as MFCC, Log-Mel, and DWT, forming a comprehensive representation of the heart sound. By combining local acoustic descriptors with global contextual embeddings, the model benefits from rich, informative features essential for accurate classification of CVD.

A key advantage of Wav2Vec is its self-supervised pretraining, which enables feature learning without labeled data by masking parts of the input and predicting them from context. The model can then be fine-tuned for domain-specific tasks, such as heart sound classification.

### 2.5. Feature Normalization

Given the diverse range of acoustic features extracted, the resulting feature vectors exhibit varying scales and dynamic ranges. To prevent features with larger magnitudes from dominating the learning process, particularly during the gradient descent optimization of the Hybrid CNN-LSTM model, we applied Z-score normalization to all input features. For a given feature *x*, the normalized value *z* is calculated as:

(14) $$z\;=\;\frac{x-\mu }{\sigma }$$

where μ and σ are respectively the mean and standard deviation calculated exclusively on the training set. These statistics were then applied to transform the Validation and Test sets to prevent data leakage.

### 2.6. Pattern Recognition Architectures

The proposed framework employs two well-known models: a CNN–LSTM hybrid model and XGBoost. Each model was trained with two different sets of features, resulting in four combinations, as detailed below.

#### 2.6.1. CNN–LSTM Hybrid Model

**Figure 2:**
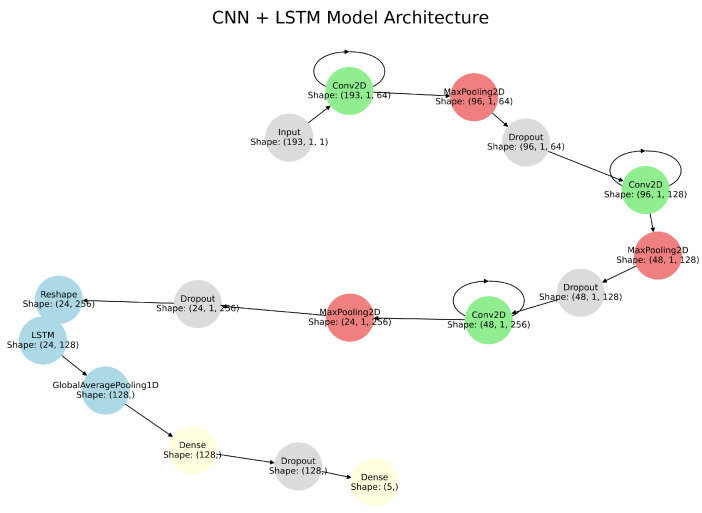
CNN-LSTM Hybrid Model Architecture showing the integration of convolutional layers for spatial feature extraction and LSTM layers for temporal analysis.

The hybrid model combines CNN and LSTM networks to exploit their complementary strengths. CNNs capture spatial features of heart sound data through convolutional layers, max-pooling layers, and dropout layers [18]. Convolutional layers apply kernels to the input data to detect frequency and intensity patterns [28]. Max-pooling layers extract the most prominent features by selecting maximum values within pooling windows, while dropout layers prevent overfitting by randomly deactivating neurons during training [29]. The extracted spatial features are then passed to LSTM layers, which model temporal dependencies and capture sequential trends over the cardiac cycle. By integrating CNN-based spatial representations with LSTM-based temporal analysis, the hybrid architecture effectively models both spectral and temporal characteristics of heart sounds (see Figure 2). Table 1 provides the detailed architecture of the CNN–LSTM model.

#### 2.6.2. XGBoost

**Figure 3:**
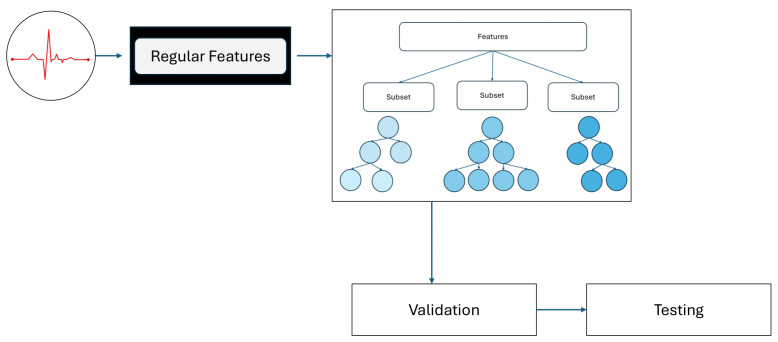
XGBoost Model Architecture showcasing key stages such as Feature Engineering, Tree Building, Gradient Boosting, Regularization, and Prediction. The model integrates multiple decision trees to create a robust ensemble learning framework for heart sound classification.

XGBoost is a highly efficient and scalable ML algorithm particularly suited for structured feature data. It employs ensemble learning by combining many weak learners (decision trees) into a strong predictor [19]. Each subsequent learner focuses on correcting the errors of the previous ones, thereby improving classification performance. XGBoost incorporates advanced regularization techniques (both L1 and L2), where L1 encourages feature selection and L2 helps prevent overfitting [19]. Beyond regularization, XGBoost handles missing values automatically, optimizes memory usage, and supports parallel and GPU-accelerated training, making it suitable for large datasets [20,30]. When trained on well-structured features obtained from feature fusion (e.g., MFCC, Log-Mel, and others), XGBoost can form a solid classification framework by integrating multiple aspects of the target signal (see Figure 3). Its ability to assign feature importance further aids interpretability, while its scalability ensures robust performance in both offline and real-time applications where speed and accuracy are critical. For these reasons, XGBoost remains one of the most powerful tools for building robust predictive models.

### 2.7. Model Configurations and Pattern Recognition Experiments

Four model configurations were evaluated in this study:

- **Wav2Vec + Hybrid Model (CNN + LSTM)**: Utilizes transformer-based Wav2Vec features in combination with a CNN + LSTM hybrid model, where CNN layers extract spatial features and LSTM layers capture temporal dependencies.
- **Wav2Vec + XGBoost**: Combines Wav2Vec features with the XGBoost algorithm, leveraging ensemble learning for robust classification.
- **Regular Features + Hybrid Model (CNN + LSTM)**: Uses traditional acoustic features (e.g., MFCC, Log-Mel, DWT) as input to the CNN + LSTM hybrid model, enabling joint spatial and temporal feature learning.
- **Regular Features + XGBoost**: Applies traditional acoustic features with XGBoost, which achieved the highest accuracy among all tested configurations.

The dataset was split into 80% for training and 20% for testing and validation. The 20% subset was further divided equally between testing and validation. The hybrid CNN + LSTM model was evaluated using K-fold cross-validation, while the XGBoost model was assessed with Monte Carlo cross-validation [31,32].

## 3. Results

Four model configurations were evaluated: (i) Wav2Vec + Hybrid Model (CNN + LSTM), (ii) Wav2Vec + XGBoost, (iii) Regular Features + Hybrid Model, and (iv) Regular Features + XGBoost. Their performances were compared based on confusion matrices and classification metrics.

### 3.1. Wav2Vec + Hybrid Model

**Figure 4:**
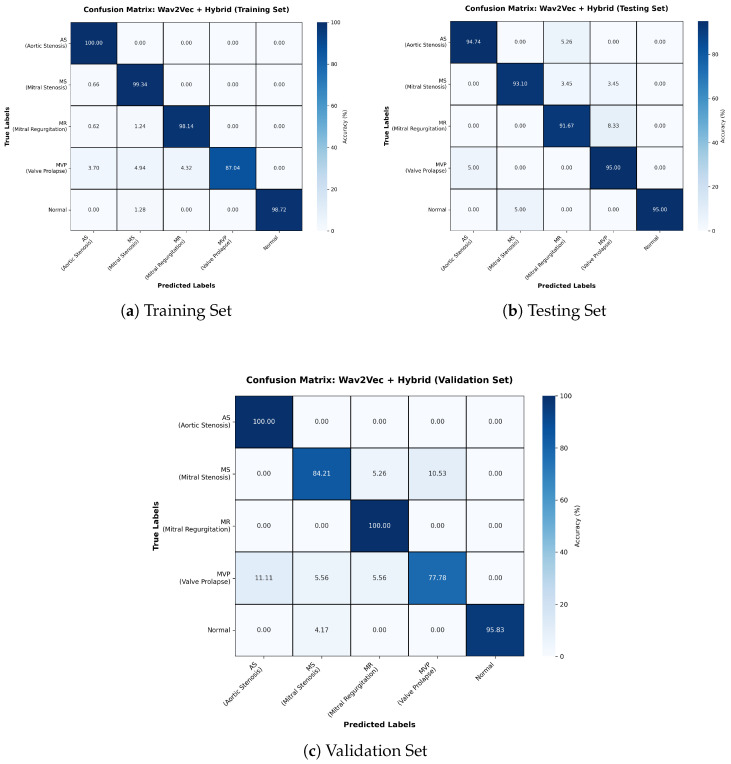
Confusion matrices for Training, Testing, and Validation sets for Wav2Vec + Hybrid model.

The Wav2Vec + Hybrid model achieved an overall accuracy of 94% (Table 2). The confusion matrices (Figure 4) show strong classification performance across all datasets, with minor misclassifications. The main challenges were observed in distinguishing MR and MVP, with MR obtaining the lowest F1-score (0.85). Although the model demonstrates balanced performance across most classes, these results suggest the need for refinement to reduce misclassification in more complex categories.

### 3.2. Wav2Vec + XGBoost

**Figure 5:**
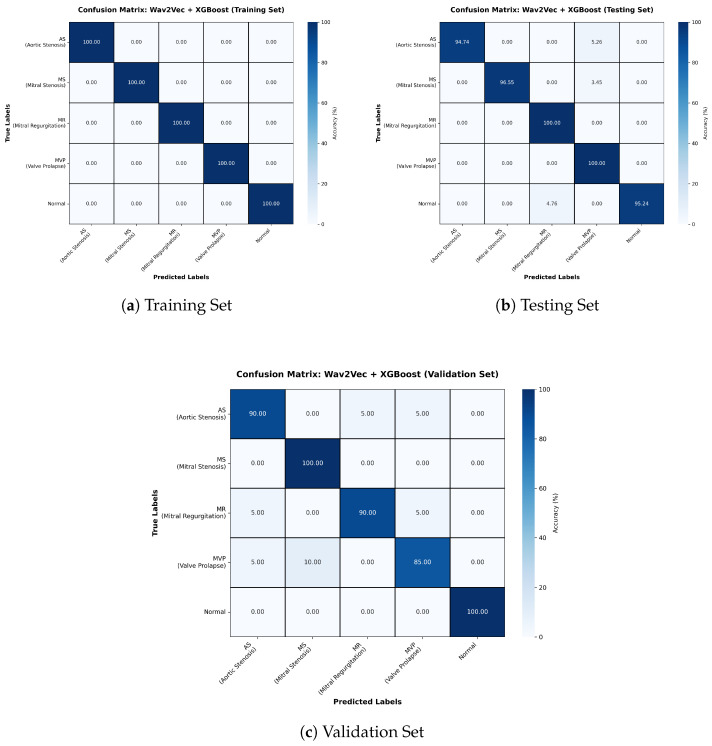
Confusion matrices for Training, Testing, and Validation sets for Wav2Vec + XGBoost model.

The Wav2Vec + XGBoost configuration also achieved an accuracy of 94% (Table 3). The training confusion matrix shows perfect predictions, but testing and validation reveal clear overfitting (Figure 5). While precision was consistent across classes, misclassifications were evenly distributed rather than concentrated in specific categories, suggesting limited generalization despite strong training performance.

### 3.3. Regular Features + Hybrid Model

**Figure 6:**
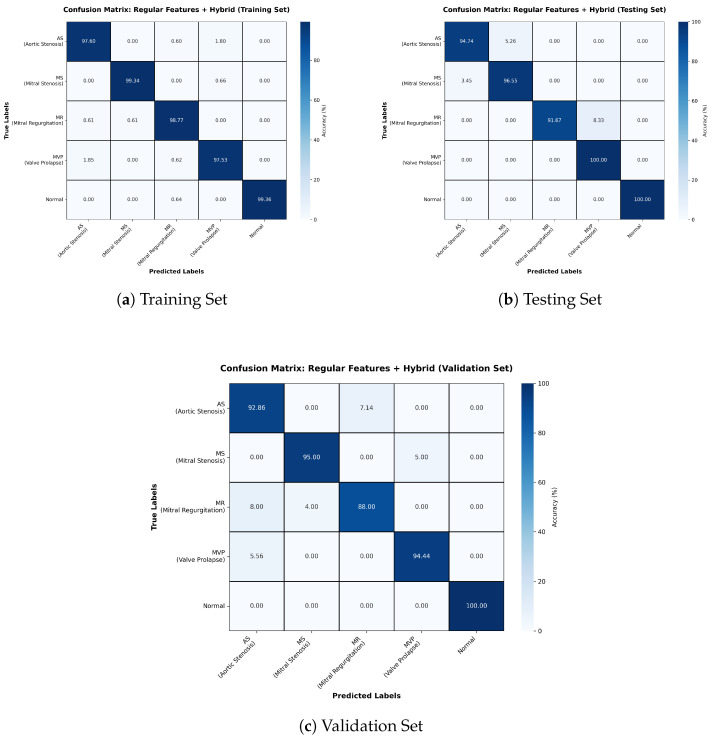
Confusion matrices for Training, Testing, and Validation sets for Regular Features + Hybrid model.

The Regular Features + Hybrid model outperformed the Wav2Vec-based configurations, reaching 98% accuracy (Table 4). Confusion matrices (Figure 6) reveal minimal misclassifications across datasets, with MR and Normal classes showing perfect precision and recall. The consistently high F1-scores underscore the reliability of this model in clinical contexts, where minimizing false negatives is critical.

### 3.4. Regular Features + XGBoost

**Figure 7:**
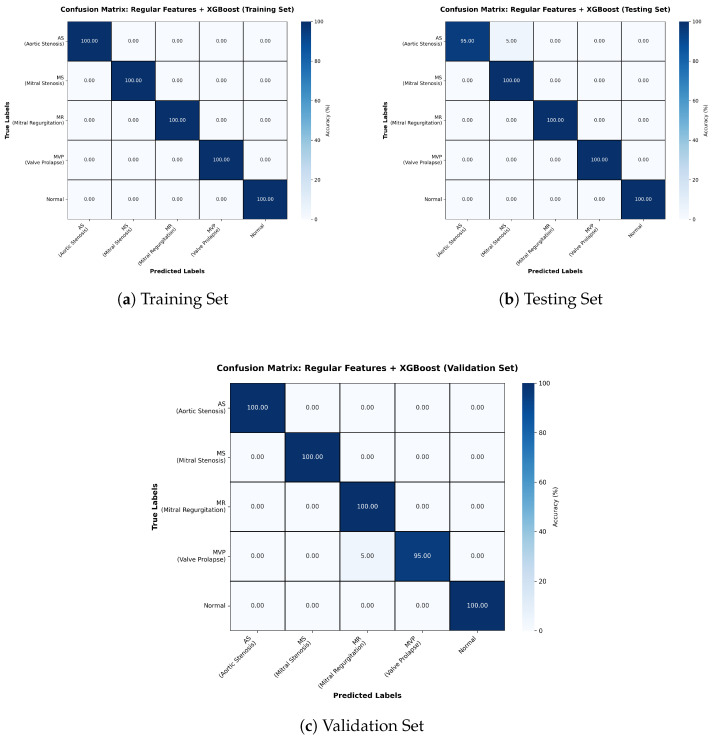
Confusion matrices for Training, Testing, and Validation sets for Regular Features + XGBoost model.

The best performance was achieved by the Regular Features + XGBoost configuration, with an accuracy of 99% (Table 5). Confusion matrices (Figure 7) confirm near-perfect classification, with only isolated misclassifications in the testing and validation sets. The model benefits from XGBoost’s ability to handle structured features efficiently, leveraging gradient boosting to combine weak learners into a highly accurate final model.

### 3.5. Comparison of Model Performance

Table 6 summarizes the test accuracies of all models. Results show that models trained on traditional acoustic features outperform those relying solely on Wav2Vec embeddings. Furthermore, XGBoost consistently outperforms the hybrid CNN + LSTM model, demonstrating superior ability to leverage structured input features efficiently.

**Figure 8:**
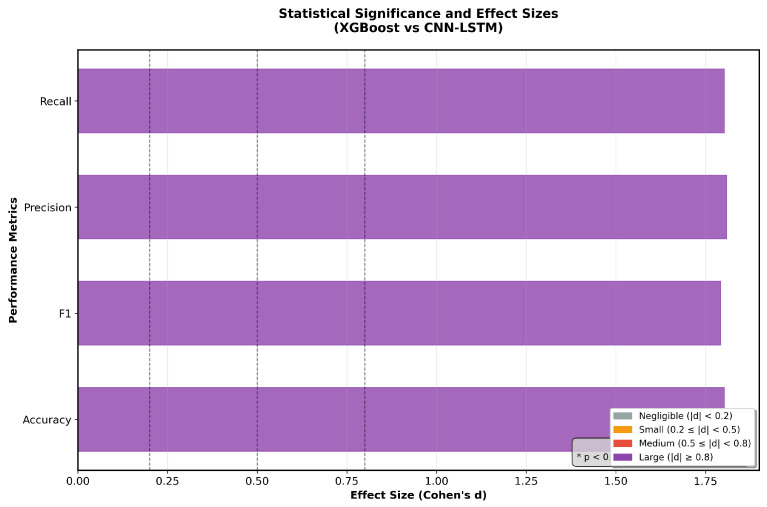
Statistical comparison of XGBoost vs. the CNN-LSTM classifier in terms of accuracy, F1-score, precision, and recall, based on Cohen’s d effect sizes.

The statistical analysis performed using pairwise rank-sum tests across the four evaluation metrics yielded *p*-values below 0.05 in all cases, indicating a statistically significant performance advantage of XGBoost over the deep learning architecture considered in this study. Beyond statistical significance, the magnitude of this difference was further assessed using Cohen’s d effect size. As illustrated in Figure 8, all metrics exhibit large effect sizes (d > 0.8), demonstrating that the observed performance gains are not only statistically meaningful but also practically relevant. These results indicate that XGBoost consistently outperforms the CNN–LSTM model with a substantial margin across accuracy, precision, recall, and F1-score, highlighting its robustness and reliability. From a clinical perspective, such improvements in classification performance can be particularly impactful, as even modest increases in diagnostic consistency may translate into enhanced confidence and effectiveness in heart sound–based disease detection.

## 4. Discussion

Heart sound classification for CVD detection remains a critical challenge in clinical diagnostics, where accurate and reliable automated methods can significantly improve patient outcomes [33]. In this study, we investigated the effectiveness of combining carefully engineered acoustic features with advanced machine learning models, i.e., XGBoost and a hybrid CNN + LSTM architecture, for multi-class classification of five heart sound categories typically associated with pathological alterations in cardiac function. Our results highlight that both the choice of features and the underlying model architecture critically influence classification performance. Models based solely on transformer-derived Wav2Vec embeddings achieved moderate accuracy, i.e., 94% for both the Wav2Vec + Hybrid and Wav2Vec + XGBoost models, demonstrating that generic speech representations, while useful, may not fully capture the unique acoustic patterns inherent in heart sounds. Despite this, Wav2Vec embeddings still offer a promising foundation, particularly when combined with complementary feature sets or fine-tuned on larger heart sound datasets. In contrast, models using traditional acoustic features, i.e., MFCC, Log-Mel, DWT, and Chroma, consistently outperformed Wav2Vec-based models, with the Regular Features + Hybrid configuration achieving 98.2% accuracy and the Regular Features + XGBoost reaching 99%. These findings indicate that domain-specific acoustic features provide richer, more discriminative information for heart sound classification than general-purpose embeddings, emphasizing the continued importance of careful feature engineering for physiological signals. A possible explanation for the comparatively lower performance of Wav2Vec-based models lies in the way transformer-derived representations are adapted for downstream classification. Wav2Vec 2.0 produces high-dimensional, frame-level contextual embeddings that preserve rich temporal information through self-attention mechanisms [22]. In this study, these representations were flattened to obtain fixed-length vectors compatible with conventional classifiers, rather than summarized using temporal pooling strategies such as mean or attention pooling. While flattening preserves fine-grained temporal detail, it also leads to very high-dimensional feature spaces, which may dilute discriminative patterns and increase sensitivity to noise when applied to relatively short and structured physiological signals such as heart sounds. In contrast, pooling-based approaches explicitly aggregate temporal information and have been shown to improve robustness in speech-related tasks by emphasizing salient temporal statistics [34]. These findings suggest that, although Wav2Vec embeddings provide a powerful representation framework, their direct application to heart sound classification may require task-specific temporal aggregation or fine-tuning strategies to better align with the quasi-periodic nature of cardiac acoustic signals.

Table 7 summarizes the comparative performance of our proposed models against related works in the literature. Previous studies, i.e., SVM, KNN, and high-frequency marker-based methods, reported accuracies ranging from 92% to 97.9%, whereas segmentation-free CNN and RNN architectures achieved 96–96.7%. This comparison demonstrates that combining multiple traditional features with robust models like XGBoost provides a measurable advantage over both conventional ML and DL architectures trained on raw or pre-trained embeddings. Notably, XGBoost leverages an ensemble of weak learners to optimize classification on structured feature sets, enabling both high accuracy and robust generalization. Simultaneously, the hybrid CNN + LSTM model exploits the complementary strengths of spatial feature extraction through CNN layers and temporal sequence modeling via LSTM layers, capturing long-term dependencies across cardiac cycles, i.e., S1, S2, S3, and S4 sounds, which are crucial for detecting subtle abnormalities.

The comparison in Table 7 also emphasizes the importance of feature selection and engineering. Our study confirms that combining multiple acoustic features enhances model performance, improving the robustness and discriminative power of both hybrid and ensemble models. The superior accuracy of the Regular Features + XGBoost model reflects the ability of gradient-boosted ensemble learning to capture complex interactions among features while mitigating overfitting and handling class imbalances effectively. This contrasts with Wav2Vec-based models, which, although leveraging transformer embeddings, may not fully capture the nuanced temporal and spectral characteristics of heart sounds. The hybrid model, by integrating LSTM layers, addresses some of these limitations by encoding temporal dynamics across sequential cardiac cycles, which is particularly beneficial for capturing irregular rhythms and murmurs.

Overall, the results highlight that a strategic combination of multiple feature extraction techniques and well-designed model architectures yields the most accurate heart sound classification. The proposed Regular Features + XGBoost model, achieving 99% accuracy, outperforms all other configurations, including both hybrid DL approaches and previously reported methods, as shown in Table 7. Beyond classification performance, a key distinction between XGBoost and deep learning architectures lies in computational efficiency and deployment feasibility. Tree-based ensemble models such as XGBoost have been shown to achieve strong performance on structured feature sets with lower computational cost and reduced memory requirements compared to deep learning models, particularly on tabular or engineered data commonly used in medical applications [35]. This efficiency is enabled by multi-core CPU parallelism, histogram-based split finding, and built-in regularization mechanisms that support robust generalization without extensive preprocessing. In contrast, hybrid CNN–LSTM architectures typically involve a larger number of trainable parameters and rely on iterative backpropagation over convolutional and recurrent layers, resulting in higher computational demands and a stronger reliance on GPU acceleration, especially when processing time–frequency representations [36]. Although a real-time evaluation of the architecture was beyond the aim of the study, the presented characteristics of XGBoost make it particularly suitable for embedded heart sound classification systems, where low latency and limited computational resources are critical.

Despite the high accuracy achieved by the Regular Features + XGBoost model, some practical barriers to clinical translation must be addressed. The current study utilized recordings obtained under controlled conditions; however, real-world clinical environments introduce significant ambient noise, patient movement, and respiratory interference that can distort the discriminative power of acoustic features [37]. Future work should focus on integrating adaptive denoising techniques or training on noisy datasets to ensure robust performance in chaotic hospital settings. Furthermore, while this model was validated on a specific electronic stethoscope, the variability in frequency responses across different digital sensors and smartphone microphones presents a challenge for generalizability [38]. Validating the model across diverse hardware platforms is essential to ensure that engineered features like MFCCs and DWT remain stable across different devices. Finally, clinical populations exhibit a significant class imbalance where normal heart sounds are orders of magnitude more common than pathological murmurs. Transitioning this technology to the bedside will require further validation on large-scale, multicenter, and uncurated datasets to verify that the model maintains its high specificity and sensitivity across diverse patient demographics [21,39].

**Table 7:** Comparison of Our Models Against Related Works.

Model	Accuracy
DNN [5]	92.10%
Wav2Vec + Hybrid Model	94.10%
Wav2Vec + XGBoost	94.40%
CNN without Segmentation [40]	96%
RNN [41]	96.70%
Centroid Displacement based KNN [5]	97.40%
High Frequency Marker [42]	97.50%
SVM [5]	97.90%
Regular Features + Hybrid Model	98.20%
Regular Features + XGBoost	99%

## 5. Conclusions

This study demonstrates that combining carefully engineered acoustic features with advanced ML models enables highly accurate heart sound classification for CVD detection. Traditional features, including MFCC, Log-Mel, DWT, and Chroma, provide rich temporal and spectral information that outperforms generic transformer embeddings like Wav2Vec. While the hybrid CNN + LSTM model captures spatial and temporal patterns effectively, XGBoost achieves superior performance in this study, reaching 99% accuracy with Regular Features compared to 98.2% for the hybrid model. This highlights XGBoost’s strength in leveraging structured multi-feature data, handling complex interactions, mitigating overfitting, and providing robust generalization. Overall, these results confirm that combining domain-specific features with ensemble learning produces a reliable, accurate, and clinically applicable solution for automated cardiovascular diagnostics. 

## Figures and Tables

**Table 1:** CNN-LSTM Model Architecture.

Layer Type	Layer Details	Input Dimensions	Output Dimensions
Input	Input Layer	100, 193, 1	100, 193, 1
Conv2D	Filters = 64, Kernel Size = (3, 1), Activation = ReLU, Padding = Same	100, 193, 1	100, 193, 64
Batch Normalization	-	100, 193, 64	100, 193, 64
Conv2D	Filters = 64, Kernel Size = (3, 1), Activation = ReLU, Padding = Same	100, 193, 64	100, 193, 64
Batch Normalization	-	100, 193, 64	100, 193, 64
MaxPooling2D	Pool Size = (3, 1)	100, 193, 64	33, 193, 64
Dropout	Rate = 0.5	33, 193, 64	33, 193, 64
Conv2D	Filters = 128, Kernel Size = (3, 1), Activation = ReLU, Padding = Same, Regularization = L2 (0.01)	33, 193, 64	33, 193, 128
Batch Normalization	-	33, 193, 128	33, 193, 128
Conv2D	Filters = 128, Kernel Size = (3, 1), Activation = ReLU, Padding = Same	33, 193, 128	33, 193, 128
Batch Normalization	-	33, 193, 128	33, 193, 128
MaxPooling2D	Pool Size = (2, 1)	33, 193, 128	33, 193, 128
Dropout	Rate = 0.5	33, 193, 128	33, 193, 128
Conv2D	Filters = 256, Kernel Size = (3, 1), Activation = ReLU, Padding = Same	33, 193, 128	33, 193, 256
Batch Normalization	-	33, 193, 256	33, 193, 256
Conv2D	Filters = 256, Kernel Size = (3, 1), Activation = ReLU, Padding = Same	33, 193, 256	33, 193, 256
Batch Normalization	-	33, 193, 256	33, 193, 256
Conv2D	Filters = 256, Kernel Size = (3, 1), Activation = ReLU, Padding = Same	33, 193, 256	33, 193, 256
Batch Normalization	-	33, 193, 256	33, 193, 256
MaxPooling2D	Pool Size = (2, 1)	33, 193, 256	16, 193, 256
Dropout	Rate = 0.5	16, 193, 256	16, 193, 256
Reshape	Reshape for LSTM	16, 193, 256	16, 49,376
LSTM	Units = 128, Return Sequences = True, Dropout = 0.5	16, 49,376	16, 128
Global Max Pooling 1D	-	16, 128	128
Dense	Units = 128, Activation = ReLU	128	128
Dropout	Rate = 0.5	128	128
Dense (Output)	Units = num_classes, Activation = Softmax	128	5

**Table 2:** Classification Report for Wav2Vec + Hybrid Model.

Class	Precision	Recall	F1 Score
Aortic Stenosis	0.95	0.95	0.95
Mitral Stenosis	1.00	0.93	0.96
Mitral Regurgitation	0.79	0.92	0.85
Mitral Valve Prolapse	0.90	0.99	0.99
Normal	1.00	0.95	0.97
Accuracy			0.94

**Table 3:** Classification Report for Wav2Vec + XGBoost Model.

Class	Precision	Recall	F1 Score
Aortic Stenosis	1.00	0.90	0.95
Mitral Stenosis	0.90	0.95	0.93
Mitral Regurgitation	0.90	0.95	0.93
Mitral Valve Prolapse	0.90	0.90	0.90
Normal	1.00	1.00	1.00
Accuracy			0.94

**Table 4:** Classification Report for Regular Features + Hybrid Model.

Class	Precision	Recall	F1 Score
Aortic Stenosis	1.00	0.95	0.97
Mitral Stenosis	1.00	0.97	0.98
Mitral Regurgitation	1.00	1.00	1.00
Mitral Valve Prolapse	0.91	1.00	0.95
Normal	1.00	1.00	1.00
Accuracy			0.98

**Table 5:** Classification Report for Regular Features + XGBoost.

Class	Precision	Recall	F1 Score
Aortic Stenosis	1.00	0.95	0.97
Mitral Stenosis	1.00	1.00	1.00
Mitral Regurgitation	0.95	1.00	0.98
Mitral Valve Prolapse	1.00	1.00	1.00
Normal	1.00	1.00	1.00
Accuracy			0.99

**Table 6:** Test accuracy of All Models.

Approach	Accuracy (%)
Wav2Vec + Hybrid Model	94.0
Wav2Vec + XGBoost	94.0
Regular Features + Hybrid Model	98.0
Regular Features + XGBoost	99.0

## Data Availability

The dataset used in this study was developed and made publicly available by Yaseen Khan.
